# Improved Classification of Alzheimer's Disease Data via Removal of Nuisance Variability

**DOI:** 10.1371/journal.pone.0031112

**Published:** 2012-02-13

**Authors:** Juha Koikkalainen, Harri Pölönen, Jussi Mattila, Mark van Gils, Hilkka Soininen, Jyrki Lötjönen, for the Alzheimer's Disease Neuroimaging Initiative

**Affiliations:** 1 VTT Technical Research Centre of Finland, Tampere, Finland; 2 Department of Neurology, University of Eastern Finland, Kuopio, Finland; 3 Department of Neurology, Kuopio University Hospital, Kuopio, Finland; University of Illinois-Chicago, United States of America

## Abstract

Diagnosis of Alzheimer's disease is based on the results of neuropsychological tests and available supporting biomarkers such as the results of imaging studies. The results of the tests and the values of biomarkers are dependent on the nuisance features, such as age and gender. In order to improve diagnostic power, the effects of the nuisance features have to be removed from the data. In this paper, four types of interactions between classification features and nuisance features were identified. Three methods were tested to remove these interactions from the classification data. In stratified analysis, a homogeneous subgroup was generated from a training set. Data correction method utilized linear regression model to remove the effects of nuisance features from data. The third method was a combination of these two methods. The methods were tested using all the baseline data from the Alzheimer's Disease Neuroimaging Initiative database in two classification studies: classifying control subjects from Alzheimer's disease patients and discriminating stable and progressive mild cognitive impairment subjects. The results show that both stratified analysis and data correction are able to statistically significantly improve the classification accuracy of several neuropsychological tests and imaging biomarkers. The improvements were especially large for the classification of stable and progressive mild cognitive impairment subjects, where the best improvements observed were 6% units. The data correction method gave better results for imaging biomarkers, whereas stratified analysis worked well with the neuropsychological tests. In conclusion, the study shows that the excess variability caused by nuisance features should be removed from the data to improve the classification accuracy, and therefore, the reliability of diagnosis making.

## Introduction

Alzheimer's disease (AD) is the most general type of dementia. It is a neurodegenerative disease that causes atrophy in the cerebral cortex and subcortical structures, such as the hippocampus and amygdala. The latest research guidelines for the diagnosis of probable Alzheimer's disease require a presence of both impairment in episodic memory and one supportive feature, either medial temporal lobe atrophy, abnormal cerebrospinal fluid biomarker, specific pattern in positron emission tomography or proven AD autosomal dominant mutation [1]–[2]. Mild cognitive impairment (MCI) is a condition in which a patient has memory impairment but activities of daily living are preserved [3]. It is a risk factor for AD, but not every MCI patient develops into AD.

Lots of interest has been recently focused on personalized healthcare or medicine [4]–[6], where the population is divided into sub-groups based on some personal factors, and the diagnosis and/or treatment is decided using the information tailored specifically to each sub-group. Typically, the sub-groups are determined based on age, gender, or genome. Considering the diagnosis of AD, demographic information of a patient, such as age, gender, education, weight, and genome have been reported to interact with the results of neuropsychological tests and biomarkers [7]–[10]. Consequently, personalized models are needed to improve the diagnosis of AD.

In statistics, such variables that affect the analysis results but are not of immediate interest are called as nuisance variables/features/covariates. In this paper, methods to remove the effects of nuisance features are studied. The methods studied benefit personalized healthcare and medicine by producing optimally personalized data that a clinician can utilize in the decision making or that can produce more accurate classifications with automated machine learning methods.

There are several ways to remove the data variability caused by nuisance features. In clinical studies, the effects of personal factors are often removed by using age- and gender-matched study groups. A similar approach is to divide the data into more homogeneous sub-groups (stratified analysis) [11]. For example, one may make separate analyses for males and females, or divide data into age groups, or do both. The nuisance data variability should be smaller in these sub-groups, and the disease-related variability should be more pronounced. In this paper, the term *data stratification* is used to refer to a method where a subset of samples is selected for the analysis based on the values of one or many nuisance features. This is the typical approach used in personalized healthcare and medication.

In statistics, analysis of covariance (ANCOVA) can be used to remove the effects of nuisance features from the independent variables (classification features, i.e., features used to classify the subjects) by means of multiple linear regressions [12]. In this study, a method related to ANCOVA is used to remove the effects of nuisance features from the classification features prior classification using a linear regression model. The term *data correction* is used to refer to this method. In some studies, the nuisance features are given as ordinary variables to the classifier, such as support vector machine, and it is assumed that the classifier is able to correct the interactions between nuisance features and classification features [13]. Other methods to eliminate the nuisance features are presented in [14], from which the most generally used methods are based on Bayesian models.

In this paper, the methods to remove the interactions of nuisance features and classification features were compared using a large dataset and a large number of classification features from several neuropsychological tests and a number of imaging biomarkers. Three methods were tested. First, in data stratification the data were divided into subgroups based on one or several nuisance features. Second, in data correction the interactions of the nuisance features were removed from the classification features using a linear regression model. Our goal was to remove the effects of nuisance features but at the same time keep all the disease-related data variability. Third method studied, which, to the best of our knowledge has not been studied previously on medical data, was the combination of these two methods. The evaluation of the methods was based on classification results of an automatic machine learning method (sequential stepwise feature selection and regression classifier). In clinical decision support systems, the knowledge obtained in this study can be used to generate data, either in textual or graphical format, where the differences between study groups are pronounced.

The objective of this paper was to study the methods for removing the effects on nuisance features from the classification data in order to get the optimal gain for the personalized healthcare and medicine. The example application for which the methods were validated was the diagnosis of AD. The results show that significant improvements in the classification of AD data are obtained using the methods studied.

## Materials and Methods

### Subjects

Data used in the preparation of this article were obtained from the Alzheimer's Disease Neuroimaging Initiative (ADNI) database (adni.loni.ucla.edu). The ADNI was launched in 2003 by the National Institute on Aging (NIA), the National Institute of Biomedical Imaging and Bioengineering (NIBIB), the Food and Drug Administration (FDA), private pharmaceutical companies and non-profit organizations, as a $60 million, 5-year public-private partnership. The primary goal of ADNI has been to test whether serial magnetic resonance imaging, positron emission tomography, other biological markers, and clinical and neuropsychological assessment can be combined to measure the progression of mild cognitive impairment and early Alzheimer's disease. Determination of sensitive and specific markers of very early AD progression is intended to aid researchers and clinicians to develop new treatments and monitor their effectiveness, as well as lessen the time and cost of clinical trials. The Principal Investigator of this initiative is Michael W. Weiner, MD, VA Medical Center and University of California – San Francisco. ADNI is the result of efforts of many co- investigators from a broad range of academic institutions and private corporations, and subjects have been recruited from over 50 sites across the U.S. and Canada. The initial goal of ADNI was to recruit 800 adults, ages 55 to 90, to participate in the research – approximately 200 cognitively normal older individuals to be followed for 3 years, 400 people with MCI to be followed for 3 years and 200 people with early AD to be followed for 2 years.

In the ADNI database, the subjects are classified into three groups: healthy controls, MCI subjects, and AD subjects. There is also follow-up information on the conversion events (MCI to AD, Control to MCI or AD). For this study, four groups were established: controls (C), stable MCIs (SMCI), progressive MCIs (PMCI), and subjects with Alzheimer's disease (AD). The SMCI group consisted of the subjects with MCI at baseline and no known conversion to AD. The PMCI subjects had MCI at baseline, and known conversion to AD during the study time. The total number of subjects was 786. The demographics of the subjects are shown in Table 1.

**Table 1:** Demographic data (mean ± standard deviation, or %) of subjects.

	C	SMCI	PMCI	AD	Total
N	217	222	156	191	786
Age (years)	76.0±5.1	75.1±7.6	74.6±6.9a	75.4±7.5	75.3±6.8
Females (%)	48.30%	33.3%^a^	40.40%	47.6%^b^	42.40%
Education (years)	16.1±2.8	15.6±3.2	15.7±2.9	14.7±3.1^a^^,^ b ^,^ c	15.6±3.1
MMSE	29.1±1.0	27.3±1.8^a^	26.7±1.7^a^^,^ b	23.3±2.1^a^^,^ b ^,^ c	26.7±2.7
Conversion time (months)			18.2±9.0		

a statistically significantly different from controls, p<0.05.

b statistically significantly different from SMCI, p<0.05.

c statistically significantly different from PMCI, p<0.05.

t-test for age, education and MMSE, chi-square test for percentage of females.

### Features

There are about 3000 features in the ADNI database. These features include, for example, demographic data, results of neuropsychological tests, molecular tests, and imaging studies. In this study, we selected nine feature groups for the analysis. A set of neuropsychological tests (both raw scores of individual tests and total scores) and biomarkers from imaging studies (original data, not normalized for brain size), which were known to be among the best ones for the classification of AD data, were selected. Also, the APOE genotype (number of APOE a4 alleles) was used. The feature groups are listed in Table 2. In order to focus on the most interesting features and features for which there are not many missing values, a pre-selection of the features was performed. A feature was excluded, if there was more than 10% values missing, and if the p-value of the t-test (data were assumed to be normally distributed) for the two study groups was above a threshold. This threshold was 0.00001 for C vs. AD comparison and 0.01 for SMCI vs. PMCI comparison. The values were chosen so that approximately as many classification features were used in both classification studies. The numbers of features in each feature group after the pre-selections are shown in Table 2. The data used in this study were the baseline features (either screening or baseline measurements).

**Table 2:** Feature groups used in the analysis, and the number of features after pre-selections.

Feature Group	Abbreviation	C vs. AD	SMCI vs. PMCI
ADAS Sub-Scores and Total Scores	ADAS	24	9
Clinical Dementia Rating	CDR	7	3
Functional Assessment Questionnaire	FAQ	12	10
Mini Mental State Examination	MMSE	16	2
Neuropsychological Battery	NB	40	21
Cross-Sectional FreeSurfer	FS	119	93
Derived Volumes	DV	14	13
SPM voxel based morphometry analysis	VBM	65	2
APOE e4 alleles	APOE	1	1

For the data correction and stratification, eight nuisance features were selected. The features are listed in Table 3. They are demographic data or clinical measures that can be easily obtained and that are not by themselves good classification features.

**Table 3:** Nuisance features and classification accuracies using each nuisance feature to classify all the data.

	Gender	Age	Education	Weight	Alcohol	Smoking	BPsyst	Cholesterol
C vs. AD	0.45	0.48	0.59	0.57	0.54	0.54	0.52	0.52
SMCI vs. PMCI	0.57	0.52	0.43	0.52	0.50	0.47	0.51	0.53

Education = Years of education, Alcohol = Alcohol abuse (yes/no), Smoking = Smoking (yes/no), BPsyst = Systolic blood pressure (mmHg), Cholesterol = Cholesterol (High performance).

### Data correction and stratification methods

**Figure 1:**
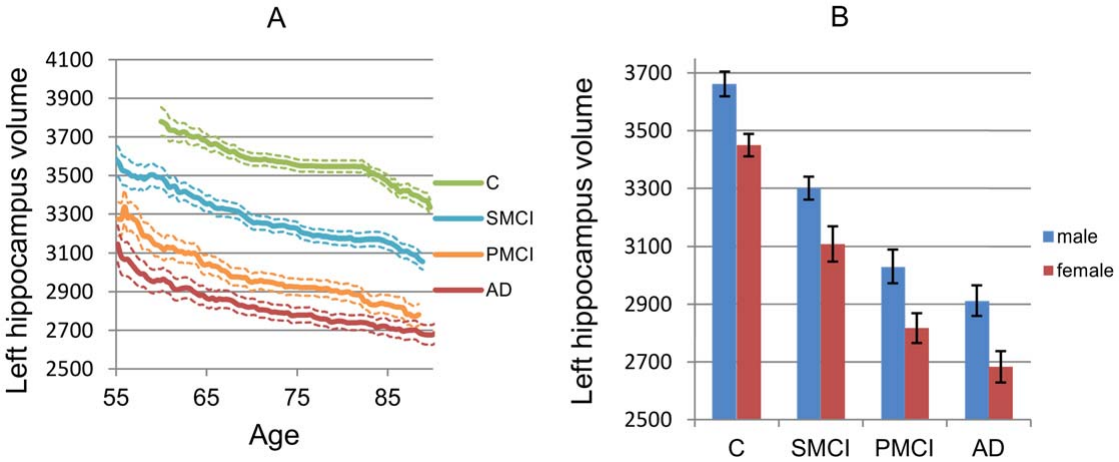
Mean left hippocampus volumes and standard errors as a function of age (males and females) and gender (for all ages).

The need for data correction or stratification methods to remove the effects of a nuisance feature is demonstrated in Figure 1, where the volume of left hippocampus is shown as a function of age and separately for males and females. In this example, there is a strong correlation between the nuisance and classification features. The average hippocampus volume of 90-years old healthy persons is close to the average volume of 55-years old AD patients. On the other hand, if the comparison is performed to the 90-years old AD subjects, the difference is evident. Therefore, by removing the age-related effect, either using data correction or data stratification, the separation of groups in the classification data is enlarged and classification accuracy is improved.

#### Data correction using regression model

As in the ANCOVA, the data correction was implemented using a linear regression model. The method was based on the data variability in the healthy controls because in this group there should not be any disease related variability, as proposed recently in [15]. The normal data variability, for example as a function of age or gender, was first modeled using regression model, and then this effect was removed from all the data using the model obtained.

Based on initial studies, we made an assumption on the linearity of the relations between the nuisance features and the classification features (e.g., Figure 1). Let us denote the value of *i*th classification feature as *c_i_*, and the value of *j*th nuisance feature as *s_j_*. Classification features are the individual features of the feature groups presented in Table 2, and the nuisance features are the features listed in Table 3.

The linear relation between classification feature *c_i_* and nuisance feature *s_j_* can be modeled using a linear regression model
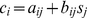
(1)where regression parameters *a_ij_* and *b_ij_* are unique for each classification–nuisance feature pair and determined as
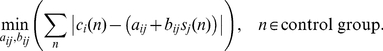
(2)Notations *c_i_(n)* and *s_j_(n)* denote the values of classification and nuisance features, respectively, for a subject *n*. The corrected values 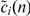 of the classification feature *c_i_* corrected for the nuisance feature *s_j_* are obtained from
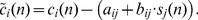
(3)The corrected values of the control group have zero mean. To produce values similar to the original values, for example, the mean value of the control group could be added to the corrected values. Alternatively, the training set used in the classification can be corrected to correspond with the values of the patient. It is straightforward to extend this method to include many nuisance features.

**Figure 2:**
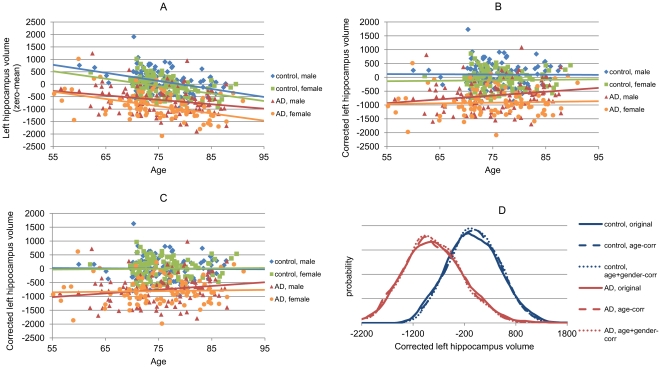
Example of data correction. Left hippocampus volume is used as the classification feature and age and gender as nuisance features. A: Original data (zero mean for visualization). B: Data corrected for age. C: Data corrected for age and gender. D: Probability distributions (Parzen widowing) for all three data. Lines visualize the linear fits for each group.

Figure 2 demonstrates the method using left hippocampus volume as the classification feature and age and gender as the nuisance features. In the original data (Figure 2A), clear trend for decreased volume in aging is observed. Also, females have lower volumes than males. First, a linear model is fit to the data of control subjects (both male and female). Then the data are corrected based on the linear model (Figure 2B), which removes most of the age-related data variability (the lines are almost horizontal). However, there are clear differences between males and females. When the correction is done by utilizing both the differences in age and gender (Figure 2C), there is no age- or gender-related variability in the control group, and also in AD group such variability has decreased notably. Consequently, the control and AD groups are better separated and the probability distributions (Figure 2D) are narrower and have higher peaks, which improves the classification accuracy.

#### Data stratification

In data stratification, a subset of the subjects is selected so that they establish a more uniform population than all the subjects together. In this study, a subset of the dataset subjects was selected for a target subject *n* based on the rule
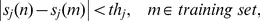
(4)where *th_j_* is a user-defined threshold for the nuisance feature *s_j_*. If multiple nuisance features are used, they all have to fulfill the rule above. The threshold *th_j_* was determined as *α*·std(*s_j_*), i.e., as a multiple of the standard deviation of the nuisance feature in the whole dataset. Different values for *α* were tested, and the results are presented for *α = 1* which gave on average the best results.

**Figure 3:**
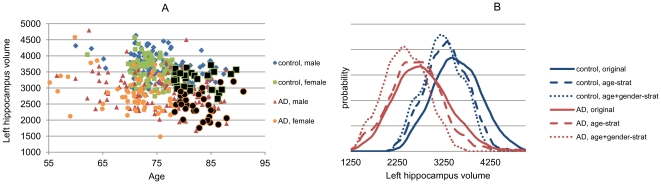
Example of data stratification. Only the subjects marked with black markers are used to train a classifier for a target subject (85 years old female).

The data stratification strategy is demonstrated in Figure 3. The example is the same as above for the data correction. In this example, it was assumed that the target subject was an 85 years old female. The subjects selected in the training set for the particular target subject are shown in black markers in Figure 3A. The probability density functions in Figure 3B show that when the stratification is done using only age, the volumes of the training sets are smaller and the probability density functions are sharper as compared to the original dataset. When the stratification is done using both age and gender the distributions are even more localized.

The differences between data stratification and data correction can be seen in the probability density functions in Figures 2D and 3B. Data stratification is able to make much larger changes in the probability distributions of the training sets. Consequently, if the target subject data match with the new distributions the data stratification should give better results. However, the number of training set samples is much smaller in the data stratification (e.g., N = 61 for data stratification in Figure 3 and N = 408 for data correction in Figure 2). This can be seen as smoother distributions in Figure 2D as compared to distributions in Figure 3B. When multiple stratification features are used, the number of training set subjects selected may be so small that a classifier, especially if high-dimensional classifiers are used, cannot be efficiently trained. One option to handle this problem would be to always use at least *N* closest samples in data stratification. However, for binary and ordinal features, such as sex, genotypes etc., such an approach is not straightforward. In this study, all the subjects were used if the number of subjects in both study groups after the data stratification was less than 10, i.e., no data stratification was performed in those cases.

#### Combination of data correction and stratification

Data correction and data stratification can be easily combined: first, data stratification is performed, and then the data selected are corrected. The data correction could be done using the stratified data only. However, as the number of samples after the stratification may be very small the estimation of the regression parameters may become inaccurate. Also, as the relationships between nuisance and classification features were assumed to be linear, the same regression parameters should be applicable for each stratified subset. Consequently, in this study we decided to make the data correction from the whole dataset in order to guarantee large enough number of samples.

### Types of interactions of nuisance and classification features

**Figure 4:**
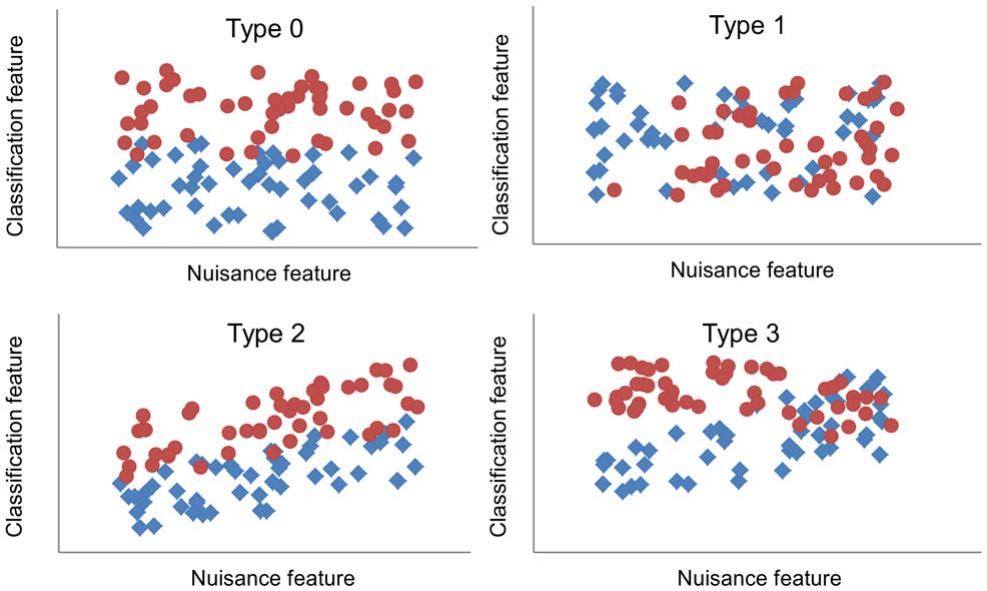
Types of relations between nuisance features and classification features.

**Figure 5:**
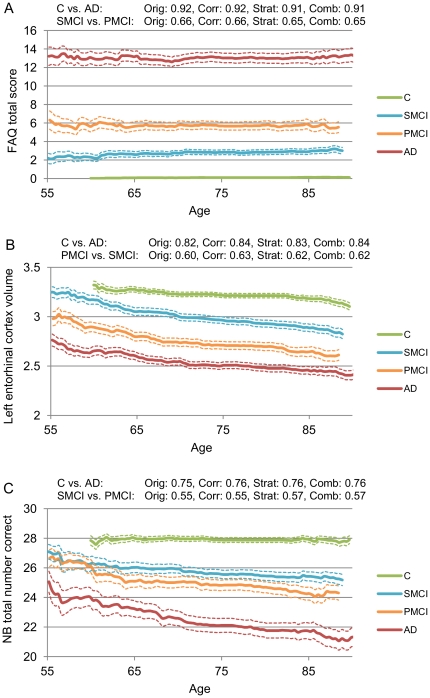
Real-world examples of the three types of interactions between a nuisance feature and a classification feature. A: Type 0. B: Type 2. C: Type 3. In the figures, the local average values and mean errors of the classification features are shown as a function of a nuisance feature. In addition, the classification accuracy for each classification feature is shown for the original data (Orig), corrected feature values (Corr), stratified analysis (Strat), and combination of data correction and stratification (Comb).

Four kinds of possible interactions between nuisance features and classification features were discovered as a result of general reasoning. These are demonstrated using synthetic examples in Figure 4 and real-world examples in Figure 5.

#### Type 0

In Type 0, there is no relation between nuisance and classification features. Therefore, neither data correction nor stratification is needed for this pair of nuisance and classification features (Figure 5A).

#### Type 1

In Type 1, there is no relation between nuisance feature and classification feature. However, the probability of belonging to a specific group is related to the value of the nuisance feature. In Figure 4, if a subject has a large value for nuisance feature, it is very likely that the subject is from the red group. In this case, the nuisance feature actually works as a classification feature, and should not be used in data correction or data stratification. No real-world example is shown for this type as the nuisance features were selected so that they cannot be used as a classification feature.

#### Type 2

If there is a direct functional relationship between the classification feature *c_i_* and the nuisance feature *s_j_* so that it can be modelled as *c_i_ = f(s_j_)*, it is possible to use either data correction or stratification to remove the effect of nuisance feature.

Theoretically, the underlying (unknown) distribution for the classification feature can be defined best by modelling the functional relationship and removing its effect from the classification feature values as in Eq 3. This way, all control subjects are used to estimate the classifier. However, in practice the underlying model can rarely be perfectly modelled and some error is introduced to the classifier. On the other hand, if stratification is used instead of modelling and a portion of the training data is left out, the expected classifier estimate would be erroneous due to the smaller sample size. Thus, it depends on the case which method is better suitable to nuisance effect correction.

As a rule of thumb, if there are a “large” number of subjects in the stratified control group or the functional relationship between the feature and the nuisance parameter is unclear, stratification should work better. With a smaller number of subjects and with an obvious functional relationship, the correction through regression modelling should be preferred. In the example in Figure 5B both the data correction and data stratification improved the classification accuracy as compared to the original feature values.

#### Type 3

In the last type of interaction, the separation of the two groups differs depending on the value of a nuisance feature. However, there is no relationship between the classification feature and the nuisance feature, so the data correction method does not work. On the other hand, data stratification can improve the classification accuracy for those nuisance feature values for which the groups are well separated. This behaviour is demonstrated in Figure 5C where especially in the classification of SMCI and PMCI subjects data stratification outperforms data correction.

When real-world data are analyzed, it is unlikely that any of the single types of interactions would be able to model all the interactions in the data. This motivates the use of the combination of data correction and stratification methods.

### Classification methods

The main objectives of this study were to compare data correction and data stratification methods, and to compare potential nuisance features to be used in data correction or stratification in the area of AD diagnosis. In order to keep the results easily interpretable, we chose to use a simple and well-known classification method, linear regression classifier. Each feature group was used separately to classify the data. Consequently, the number of features in the classification varied between one and 119 (see Table 2). Therefore, feature selection was used to avoid over-learning of classifiers.

#### Feature selection

In multi-dimensional classification, the performance of classifiers suffers if many features with little value for classification are included. The classifier tries to explain the variations in these features, and the real important information cannot be modeled accurately. Therefore, feature selection was utilized to find the optimal set of features. We used here sequential step-wise selection (Matlab's “stepwisefit” function, Matlab R2010b, The MathWorks Inc.). In the beginning, no features are in the model, and the features are added or removed one by one. The feature with the smallest p-value (if p<0.05, F-statistics) is added to the model, and if there is a feature with a p-value larger than 0.1 it is removed.

#### Regression classifier

We selected as a classifier a linear regression classifier because it is generally used and simple classifier. It is well suitable for both single-and multi-feature classification, and does not require optimization of any parameters.

There is a misbalance in the sizes of the study groups, especially between SMCI and PMCI groups (Table 1). In order to guarantee that the classifier actually classifies the data based on the classification features, not just assign the class of the largest study group to all the subjects, synthetic samples were generated from the smaller group using the SMOTE method [16] so that equal number of samples were used from both study groups.

### Evaluation procedure

In this paper, two classification studies were performed. In the first study, control subjects and AD patients were classified. This reveals basic information on the changes that take place due to the disease. However, because some feature groups, such as Mini Mental State Exam (MMSE) and Clinical Dementia Rating (CDR), are utilized in making the diagnosis, the classification results using these neuropsychological tests are biased. The second classification study was performed between stable and progressive MCI subjects. This is clinically a much more relevant study for the early detection of the disease. If the MCI subjects that will develop AD are detected early, treatments will be more effective and the costs of the disease are reduced.

The evaluation was performed using leave-N-out cross-validation: 90% of subjects were randomly selected to a training set and the remaining 10% established the test set. In data correction, the linear model was first learned from the training set, and the classifier was trained using the corrected values of the training set. Then, the classifier was applied to the corrected values of the test set subjects. In data stratification, each test set subject was individually classified by performing the stratification from the training set and training the classifier with the selected subset of the training set. This was repeated 100 times each time randomly selecting the training and test sets, and the classification results were averaged. The random training and test sets were the same for all the studies performed. Therefore, the results obtained can be pair-wise compared.

First, the classifications were performed without data correction or data stratification to establish reference results. Then all the combinations of nuisance features were studied. In other words, each nuisance feature was used first individually. Then, all the combinations of two features were studied etc. For each combination of nuisance features, the data correction, data stratification, and their combination were tested.

The evaluation was performed by measuring classification accuracy (Acc):
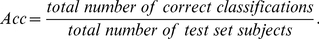
(5)Acc = 1 if all the test set subjects have been correctly classified and Acc = 0 if all have been misclassified. Statistical comparisons of classification accuracies were performed using pairwise t-test. The threshold for significance was set at *p<0.05*.

## Results

The classification results without data correction or stratification for each feature group are presented in Table 4 (column original). As assumed, the neuropsychological tests gave clearly the best results for the C vs. AD classification, with accuracy close to 100%. The best MRI-based feature group (DV) reached classification accuracy of 87%. For the classification of stable and progressive MCIs, the best results (67%) were obtained using neuropsychological test NB and the best imaging feature group was DV (65%).

**Table 4:** Classification accuracies with single nuisance feature.

C vs. AD	original	correction		stratification		combination	
ADAS	0.96	0.96	(Gender)	0.96	(Smoking)	0.96	(Smoking)
CDR	0.97	0.97	(Weight)	0.98*	(Gender)	0.98*	(Gender)
FAQ	0.96	0.96	(Cholesterol)	0.96	(Alcohol)	0.96	(Alcohol)
MMSE	0.95	0.95	(Smoking)	0.95*	(Weight)	0.95*	(Weight)
NB	0.99	0.99	(Cholesterol)	0.99	(Cholesterol)	0.99*	(Age)
FS	0.85	0.87*	(Age)	0.85	(Alcohol)	0.85	(Alcohol)
DV	0.87	0.88*	(Age)	0.87	(Alcohol)	0.87	(Age)
VBM	0.75	0.76*	(Age)	0.75	(Education)	0.76	(Education)
APOE	0.69	0.69	(Gender)	0.69	(Gender)	0.69	(Gender)

SMCI vs. PMCI	original	correction		stratification		combination	
ADAS	0.64	0.64	(Education)	0.65	(Weight)	0.65	(Weight)
CDR	0.58	0.58	(Gender)	0.63*	(Gender)	0.63*	(Gender)
FAQ	0.63	0.64	(Cholesterol)	0.65*	(Education)	0.65*	(Education)
MMSE	0.60	0.61*	(Education)	0.64*	(Gender)	0.64*	(Gender)
NB	0.67	0.68	(Cholesterol)	0.66	(Alcohol)	0.66	(Smoking)
FS	0.60	0.62*	(Age)	0.61*	(Smoking)	0.62*	(Age)
DV	0.65	0.69*	(Age)	0.67*	(Age)	0.68*	(Age)
VBM	0.51	0.56*	(Age)	0.54*	(Age)	0.56*	(Age)
APOE	0.61	0.61	(Gender)	0.61	(Age)	0.61	(Age)

Classification accuracies without data correction or stratification (original) and the best accuracies when using a single nuisance feature. The feature producing the best result is shown in parenthesis.

*statistically significantly better (p<0.05, t-test) as compared to the results with the original data.

The best results for the data correction, stratification, and their combination for single nuisance features are also shown in Table 4. In the C vs. AD classification, statistically significant improvements were observedfor all the MRI-based feature groups, in which the classification accuracy improved 1–2% units. Data stratification gave statistically significant improvement when using CDR and MMSE tests. Data stratification gave on average slightly worse results than data correction. Both methods were able to always give at least as good accuracy as the original data. The combination of data correction and data stratification did not give any statistically significant improvement, as compared to the better one of the data correction and data stratification methods alone. In the SMCI vs. PMCI classification, data correction improved the classification accuracy statistically significantly for all the MRI-based feature groups, and the improvement was 2–5% units. Data stratification improved the results of several neuropsychological tests (CDR 5% units, FAQ 2% units, and MMSE 4% units). The improvements of the MRI feature groups in data stratification were between 1–3% units. Combination of the two methods was not able to further improve classification accuracy.

Table 5 summarizes the results for the optimal combinations of nuisance features. The results for C vs. AD classification were similar to the result using only one nuisance feature. Data correction was able to improve the results of MRI-based feature groups. The improvement was 2–3% units, i.e., slightly better than with a single feature. The results of data stratification were almost similar to the results with a single nuisance feature, and the combination did not improve the results of data correction and data stratification alone. For the SMCI vs. PMCI classification, the utilization of many nuisance features improved the results more. For the MRI feature groups the improvement using data correction was 4–6% units, and for the neuropsychological tests using data stratification 2–6% units. The combination of data correction and stratification was able to give a small, but statistically non-significant, improvement to some feature groups.

**Table 5:** Classification accuracies using an optimal combination of nuisance features.

C vs. AD	original	correction		stratification		combination	
ADAS	0.96	0.96*	(Gender+Alcohol+Smoking+Cholesterol)	0.96	(Gender+Education+Alcohol)	0.96	(Smoking)
CDR	0.97	0.97*	(Gender+Weight+Alcohol+Smoking)	0.99*	(Age+Education+Weight+Alcohol+Cholesterol)	0.99*	(Age+Education+Weight+Cholesterol)
FAQ	0.96	0.96	(Cholesterol)	0.96	(Gender+Age+Education+Weight+Alcohol+Smoking+BPsyst+Cholesterol)	0.96	(Alcohol)
MMSE	0.95	0.95	(Weight+Alcohol+Smoking+BPsyst+Cholesterol)	0.95*	(Weight+Alcohol)	0.96*	(Weight+Alcohol)
NB	0.99	1.00*	(Education+Weight+Smoking+Cholesterol)	0.99	(Cholesterol)	0.99*	(Age+Alcohol)
FS	0.85	0.88*	(Gender+Age+Education+Weight+Smoking+BPsyst)	0.86	(Gender+Alcohol)	0.87*	(Gender+Age+Education+Weight+Alcohol+Smoking+BPsyst+Cholesterol)
DV	0.87	0.89*	(Gender+Age+Education+Alcohol+Smoking+Cholesterol)	0.87	(Gender+Age+Education+Smoking+BPsyst+Cholesterol)	0.88*	(Age+Education+Weight+Smoking+BPsyst+Cholesterol)
VBM	0.75	0.77*	(Gender+Age+Education+Cholesterol)	0.76*	(Education+Alcohol)	0.77*	(Gender+Age+Education+Alcohol+Smoking+BPsyst+Cholesterol)
APOE	0.69	0.69	(Gender)	0.69	(Gender+Age+Weight+Alcohol+Cholesterol)	0.69	(Gender+Age+Weight+Alcohol+Cholesterol)

SMCI vs. PMCI	original	correction		stratification		combination	
ADAS	0.64	0.65	(Age+Alcohol+Smoking)	0.66*	(Gender+Age+Education+BPsyst)	0.66*	(Gender+Education+Weight+Cholesterol)
CDR	0.58	0.58	(Alcohol+BPsyst)	0.64*	(Gender+Weight+Alcohol+Cholesterol)	0.64*	(Gender+Weight+Cholesterol)
FAQ	0.63	0.64	(Education+Weight+Alcohol+Smoking)	0.65*	(Education)	0.66*	(Education+Smoking)
MMSE	0.60	0.62*	(Gender+Age+Education+Alcohol)	0.64*	(Gender)	0.64*	(Gender)
NB	0.67	0.69*	(Gender+Age+Education+BPsyst)	0.67	(Gender+Education+Weight+BPsyst+Cholesterol)	0.68*	(Gender+Age+Education+Weight+Alcohol+Smoking+Cholesterol)
FS	0.60	0.64*	(Age+Weight+Cholesterol)	0.64*	(Alcohol+Smoking)	0.63*	(Gender+Age+Weight+Alcohol+Smoking+BPsyst+Cholesterol)
DV	0.65	0.70*	(Gender+Age+Alcohol+Smoking+BPsyst)	0.67*	(Age)	0.69*	(Gender+Age+Education+Smoking+BPsyst+Cholesterol)
VBM	0.51	0.57*	(Age+Weight+Smoking+BPsyst+Cholesterol)	0.56*	(Gender+Age+Alcohol+Smoking)	0.57*	(Gender+Age+BPsyst+Cholesterol)
APOE	0.61	0.61	(Gender)	0.62	(Gender+Education+Smoking+BPsyst)	0.62	(Gender+Education+Smoking+BPsyst)

*statistically significantly better (p<0.05, t-test) as compared to the results with the original data.

**Figure 6:**
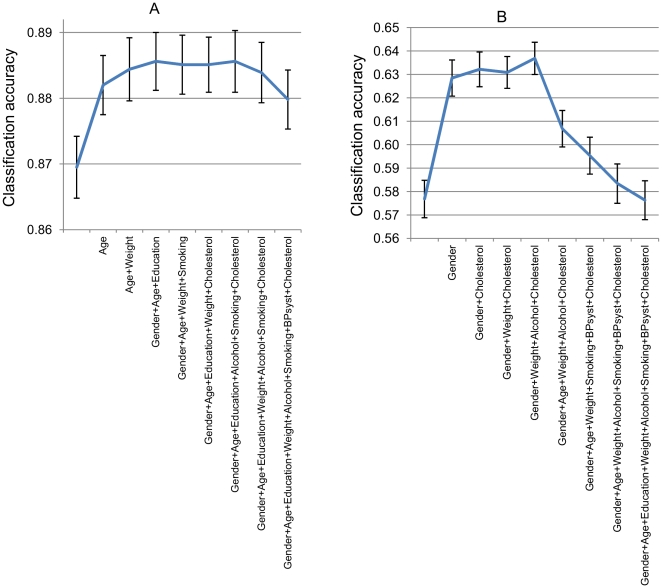
Classification accuracies and mean errors for optimal sets of *n* nuisance features. A: Data correction method applied to DV for C vs. AD classification, B: Data stratification method applied to CDR for SMCI vs. PMCI classification.

Figure 6 shows how classification accuracy changed when more nuisance features were added: it increased at first, but after reaching the optimum it began to decrease. Therefore, all the nuisance features should not be used.

The results in Tables 4 and 5 give the results for the optimal combination of nuisance features, but as these results are usually obtained with different sets of nuisance features additional guidelines are needed for future studies. Various sets of nuisance features produce results that are close to the optimal results. Consequently, it is useful to define one set of nuisance features that can be used in all the studies. However, the behaviour of neuropsychological tests and MRI biomarkers are different. Therefore, different future guidelines are needed for neuropsychological tests and imaging biomarkers. When performing classifications using imaging biomarkers, the combination of age, gender, and education in data correction gave good results for all the MRI feature groups (C vs. AD: 0.88 for FS, 0.89 for DV, 0.76 for VBM, SMCI vs. PMCI: 0.63 for FS, 0.69 for DV, 0.55 for VBM). In all these cases the results were statistically significantly improved from the classification without data correction or stratification. For the neuropsychological tests such single guideline could not be determined, but it is suggested to use the best single nuisance features reported in Table 4, i.e., gender for CDR and MMSE and education for FAQ using the combination of data correction and stratification.

To give some idea on the effect of data correction for the performance of single features, Table 6 shows the results for each feature from the DV feature group for data correction using gender, age, and education as nuisance features. Almost all the features improved the results in data correction. In the SMCI vs. PMCI classification there is a trend that the improvement is larger for the structures that give the worst results using the original data.

**Table 6:** Classification results for single features from the DV feature group.

	C vs. AD, original	C vs. AD, corrected	SMCI vs. PMCI, original	SMCI vs. PMCI, corrected
Whole Brain	0.60	0.63	0.55	0.60
Ventricles	0.65	0.67		
Right Inferior Lateral Ventricle	0.70	0.74	0.60	0.62
Left Middle Temporal	0.75	0.79	0.63	0.63
Left Inferior Lateral Ventricle	0.73	0.75	0.57	0.59
Left Hippocampus	0.79	0.82	0.61	0.64
Right Hippocampus	0.76	0.79	0.61	0.63
Left Fusiform	0.76	0.77	0.59	0.62
Right Fusiform	0.74	0.75	0.62	0.63
Right Inferior Temporal	0.74	0.75	0.64	0.65
Right Middle Temporal	0.74	0.76	0.65	0.64
Left Inferior Temporal	0.79	0.79	0.61	0.64
Left Entorhinal	0.82	0.84	0.60	0.61
Right Entorhinal	0.80	0.80	0.63	0.63

## Discussion

In this paper, data correction and data stratification were tested for the classification of Alzheimer's disease data. Eight features were used as nuisance features, and the classifications were performed using data obtained from the ADNI database. The classification data included neuropsychological tests, MRI analyses, and APOE genotype values.

The results show that in the best case up to 6% units improvement in the classification accuracy can be achieved with data correction and stratification. The biggest improvements were obtained in the classification of stable and progressive MCI subjects, which is the most challenging and interesting classification problem in the analysis of Alzheimer's disease. The classification accuracy was improved in all imaging and neuropsychological feature groups studied. In the classification of controls from AD patients, the largest improvements were obtained for the MRI-based feature groups. However, neuropsychological tests are biased in this comparison as they are already used in the clinical diagnosis, so their results are not very interesting. The data correction method gave better results for imaging biomarkers, whereas data stratification worked well with the neuropsychological tests. The combination of data correction and stratification was not able to further improve the results. Guidelines for the future studies were presented based on the results obtained in this study.

The weakness of data stratification, especially when numerous nuisance features are used, is that the number of training samples decreases which affects the performance of the classifier. This can be seen in Figure 5A, where data stratification gives worse results than the original data. On the other hand, data correction always utilizes the full data set, and, therefore, it is guaranteed that the maximum amount of data is available for the training of a classifier. In addition, data stratification requires one parameter to be chosen. The threshold *th* for the inclusion criteria was selected in this study as the standard deviation of the feature values. However, the threshold used here may not be optimal, and further studies are needed to find out how this value should be defined.

The data correction method used in this paper was based on linear regression model. We have tested also regression model with the cross-terms, but the results were worse than the results presented in this paper. The method can be easily extended to any higher order polynomials, or other basic functions. However, the more complex the model used the larger training set is required in order to reliable estimate the values for the parameters.

In this paper, we identified four types of interactions between nuisance features and classification features. Different interactions and their combinations need different correction and stratification methods. In this study, the interactions were not detected from the data, but in an optimal situation, the types of interactions are detected for each classification-nuisance feature pair, and the method used is determined based on the type detected. The types could be detected, for example, by studying the results of line fitting and statistical tests, performed either for the whole space of nuisance feature values or for a specific range of values.

The results presented in this study were obtained using linear regression classifier. All the studies were performed also using naïve Bayesian classifier, linear discriminant analysis, and support vector machines (SVM). These methods produced results similar to the ones presented in this paper. A regression classifier was selected because it is the simplest one from the classifiers studied and does not require optimization of any parameters. For example, SVM could give slightly better classification results but the choice of kernel type and parameter values should be optimized separately for each feature group used. The failure of using optimal kernels and parameters could decrease the results dramatically.

One weakness of the study may be the feature selection used. In some feature groups there were tens of features, and therefore, efficient feature selection is required. Only one standard method was tested for the feature selection in this study. A state-of-the art feature selection might give some extra improvement in the results.

There are many kinds of features in the ADNI database (continuous, binary, ordinal, nominal etc…). The methods presented here can be used for all the features that are ordered. Data stratification can be used also for non-ordered data if the thresholds are reasonably selected. The data correction method is best suitable for continuous classification features. In the case of binary or ordinal features large corrections are required to change the classification feature value so that it would affect the classification result. In this study, it was shown that data correction suits for continuous imaging biomarkers, whereas data stratification works well for neuropsychological tests where there are many binary variables. Consequently, different methods for different feature types should be further studied.

The benefit of the methods studied here is that in the clinical decision making, for example using the tool presented in [17], a clinician can visually compare the patient values to either corrected values in a dataset or to the values of a stratified dataset. In the stratified/corrected feature values the group differences are better visible and, consequently, the diagnosis can be performed more reliably. Most of the methods presented in the literature, such as ANCOVA, handle the patient values in a black box and only output a classification result that is not very informative in clinical decision making.

In this paper, the evaluation of the methods was performed using automatic classification methods in order to be able to use large dataset. An alternative approach would have been to give the original and corrected or stratified data to a clinician and asked him/her to make the diagnosis using the data available. Only a small dataset could have been evaluated using this approach, and all the combinations of nuisance features could not have been analyzed. Nevertheless, this study gave valuable information how the personalized diagnostics in AD could be performed. As the next step, this knowledge should be validated in clinical environment.

The methods proposed here can be used as a pre-processing step to improve the classification accuracy of any combination of feature selection and classification methods. In addition, the methods studied in this paper are not specific to Alzheimer's disease, but can be applied to any medical application, and also outside the medical field.
